# Automated Indoor Image Localization to Support a Post-Event Building Assessment

**DOI:** 10.3390/s20061610

**Published:** 2020-03-13

**Authors:** Xiaoyu Liu, Shirley J. Dyke, Chul Min Yeum, Ilias Bilionis, Ali Lenjani, Jongseong Choi

**Affiliations:** 1 School of Mechanical Engineering, Purdue University, West Lafayette, IN 47907, USA; liu1787@purdue.edu (X.L.); ibilion@purdue.edu (I.B.); alenjani@purdue.edu (A.L.); choi343@purdue.edu (J.C.); 2 Department of Civil and Environmental Engineering, University of Waterloo, Waterloo, ON N2L 3G1, Canada; cmyeum@uwaterloo.ca

**Keywords:** post-event building assessment, visual odometry, 3D reconstruction

## Abstract

Image data remains an important tool for post-event building assessment and documentation. After each natural hazard event, significant efforts are made by teams of engineers to visit the affected regions and collect useful image data. In general, a global positioning system (GPS) can provide useful spatial information for localizing image data. However, it is challenging to collect such information when images are captured in places where GPS signals are weak or interrupted, such as the indoor spaces of buildings. The inability to document the images’ locations hinders the analysis, organization, and documentation of these images as they lack sufficient spatial context. In this work, we develop a methodology to localize images and link them to locations on a structural drawing. A stream of images can readily be gathered along the path taken through a building using a compact camera. These images may be used to compute a relative location of each image in a 3D point cloud model, which is reconstructed using a visual odometry algorithm. The images may also be used to create local 3D textured models for building-components-of-interest using a structure-from-motion algorithm. A parallel set of images that are collected for building assessment is linked to the image stream using time information. By projecting the point cloud model to the structural drawing, the images can be overlaid onto the drawing, providing clear context information necessary to make use of those images. Additionally, components- or damage-of-interest captured in these images can be reconstructed in 3D, enabling detailed assessments having sufficient geospatial context. The technique is demonstrated by emulating post-event building assessment and data collection in a real building.

## 1. Introduction

Engineers often learn from observing the consequences of natural disasters on our physical infrastructure by studying the real world. A large amount of data is collected after each hazard event. Among the various types of data being collected, image data offer the most direct and useful way to record the impact of these events on our physical infrastructure. By utilizing inexpensive cameras or cell phones, engineers and researchers can rapidly capture and document damage or failures in a building such as spalling, shear cracks, deformation, etc. Thus, image collection is an indispensable process to support post-event building assessment. 

With the frequency of recent events and the ease with which an increasing number of images can be collected (e.g., smartphone [1,2], streetview [3,4], and aerial images [5]), the number of images being collected to support such research is growing exponentially [6,7,8,9,10,11,12]. Accordingly, it is critical to developing the capabilities associated with automatically and rapidly analyzing and publishing those data. This task has become a common goal shared across the entire hazard community. In 2016, the National Science Foundation has established a shared-use facility, the Natural Hazards Engineering Research Infrastructure (NHERI), dedicated to supporting the broad community of researchers in hazards and resilience. The RAPID facility in the NHERI network is dedicated to supporting field data collection using a variety of advanced sensors and sensing platforms [9]. A complementary component of this research network is a data repository, DesignSafe-CI, hosted at the University of Texas, United States, to document and store the data collected. Additionally, there are a couple of existing data repositories having similar purposes and functionalities, for instance through the Earthquake Engineering Research Institute, DataCenterHub, and the Pacific Earthquake Engineering Research Center in the United States, and through QuakeCore in New Zealand [10,11,12].

Despite the investments made in acquiring and storing these data, the current suite of data repositories may not be adequate for conducting in-depth research with images. Inherently, image data lack spatial context. When 3D scenes are captured with 2D images, the relative locations between the scenes on the images are needed to understand spatial relationships from 2D images [13,14,15]. To capture visual details, field engineers may take photos close to the object being documented within the scenes-of-interest. However, with such images, one cannot easily infer any of the relevant spatial contexts to make use of the information extracted. For example, assume that an engineer takes pictures of a damaged column of the building from a close distance. The images may not contain contextual information associated with, for instance, the column location on the floor, its relative size, or the relative conditions of other nearby building components. To obtain such information the engineer will need to sift through the set of previous or next pictures collected near this region. However, this is challenging, especially when one considers that a building is likely to have multiple components that are built with a common style or appearance [16]. In other words, many columns or walls in a given building do look similar to each other. In this circumstance, using images without contextual information will inevitably lead to untrustworthy results in practice, and will certainly result in needless consumption of time and manual labor involved in inferring spatial information. GPS metadata on images can provide approximate geospatial information, but only for those data captured outside the building or in open spaces without interference. Additionally, 3D sensors like Light Detection and Ranging (LiDAR) can be used for reconstructing the scenes in 3D for image localization, but they are expensive, and at this time require considerable extra time and effort to use [17,18].

To address this challenge, we have developed a technique to localize inspection images onto the structural drawings and reconstruct a local 3D textured model of scenes-of-interest. A major opportunity enabled by this technique is to achieve these capabilities without adding extra effort to or interrupting the existing data collection process in the field or without utilizing an expensive 3D sensor. In addition to the images collected for inspection (hereafter, InspImgs (inspection images)), engineers must simply collect a steady stream of images using a compact camera, potentially mounted on their hard hat or chest (hereafter, PathImgs (path images)). By implementing a visual odometry algorithm using the PathImgs, the relative locations of PathImgs along the path taken through the building being inspected are estimated, and a 3D point cloud model of the scenes is reconstructed. Structural drawings of the building may also be automatically reconstructed from drawing images (hereafter, DrawImgs (partial drawing images)) using the technique already developed by the coauthors [19]. By transforming the point cloud model to the drawing coordinates, InspImgs, which are taken at the time when PathImgs are captured, can be mapped and localized on the reconstructed drawing. Additionally, since we collect a large number of PathImgs along the path, any useful scenes on InspImgs can be reconstructed in 3D including color surface texture, enabling their detailed inspection with sufficient spatial context. To demonstrate the capability of the proposed technique, we have conducted an experiment on a building, assuming that we follow the typical procedures performed during a post-earthquake reconnaissance mission.

The remainder of this paper is organized as follows. Section 2 starts with a review of the state-of-the-art in path reconstruction techniques. In Section 3, the technical approach is described, including a detailed technical discussion of the image collection, path reconstruction, drawing reconstruction, and the path overlaid onto the drawing. In Section 4, the technique is demonstrated using the images collected by a human data collector walking through an actual building. The paper ends with the conclusion in Section 5. 

## 2. Literature Review of Path Reconstruction Techniques

A main technical challenge of our approach lies in how to recreate the path of an engineer walking through an indoor environment so that InspImgs can be mapped onto the reconstructed path. The use of GPS metadata in each image would be a logical solution, but they are extremely limited within an indoor environment [20]. Another possible technique for localizing image positions where no GPS signal exists is an indoor positioning system. Such systems adopt beacon-based methods of communicating with various signals like vision, infrared, ultrasound, Bluetooth, Wi-Fi, and radio-frequency identification (RFID) [21,22,23,24,25,26,27]. They are based on communications or measurements between mobile devices and fixed beacons that serve as landmarks. However, the preparation needed to place the necessary fixed devices before using such a system is an obvious limitation [28]. In reality, after significant disasters, buildings often have no electricity and telecommunication service is not available. Furthermore, time is tight and engineers want to collect data from several buildings each day. In typical reconnaissance procedures, it would be extremely unlikely to have the ability to set up the necessary indoor landmarks before gathering data. Therefore, beacon-based localization is infeasible in the field.

Alternatively, visual odometry (hereafter, VO) provides a potential solution that offers accurate positional output without having prior information about the environment and without relying on other sensors installed in the building [29,30]. Originating from visual-based navigation systems for mobile robots called simultaneous localization and mapping (SLAM), the technique performs the localization of a camera (including transposition and rotation movement) using a stream of still images as inputs. The accuracy and speed are improved by incorporating techniques, such as loop closure detection, map reuse, etc. [31,32,33]. In general, depending on how many cameras are engaged in the data collection process, VO can be categorized as either stereo or monocular VO [29,30]. Stereo VO, inspired by human eyes, constructs 3D depth images [34]. The main advantage of the stereo VO is that scale information of the scene can be obtained from the known distance between the lens, called, intra-axial distance. However, users need to purchase a stereo camera or manually calibrate two cameras to implement the algorithm. In contrast to stereo VO, monocular VO only uses the data from a low-cost single camera. The main disadvantage of monocular VO is that this method can only provide relative positions because there is no physical scale information available to the algorithm. In the technique described herein, the true (physical) scale information is not necessary because we only need to find the parameters needed for transforming the reference coordinate for the camera path to the coordinate for the structure drawing. Thus, we selected monocular VO to estimate the position information by collecting and processing a stream of PathImgs. 

Several successful monocular VO have been published by researchers. For instance, parallel tracking and mapping (PTAM) has improved mapping results using a new feature-based method [35], and oriented fast and rotated BRIEF SLAM (ORB-SLAM) has achieved accurate reconstruction results in a fast speed using the key frame notion [36]. Direct methods have been shown to rebuild the path and the map accurately as well, such as large-scale direct monocular SLAM (LSD-SLAM) [37]. Here, for path reconstruction, we adopted a state-of-art odometry technique called direct sparse odometry (DSO). DSO has been recognized as one of the best VO techniques with several advantages, including accuracy, processing and implementation times, etc. [38]. 

## 3. Technical Approach

**Figure 1:**
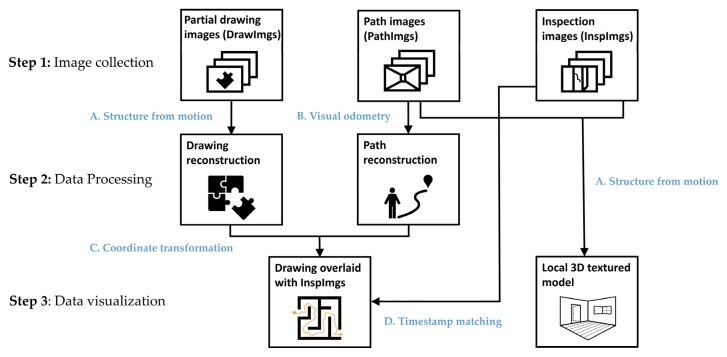
Overview of the technical approach.

An overview of the technical approach is shown in Figure 1. The technique consists of three main steps including image collection, data processing, and data visualization, and implements four algorithms (marked as A–D) into the process to generate two outcomes: a drawing overlaid with InspImgs and a local 3D textured model in Step 3. In Step 1, as the input for the proposed technique, engineers collect three types of images from the building including InspImgs, PathImgs, and DrawImgs. InspImgs are the data being collected in the field, aiming to capture buildings and their components for the purposes of visual assessment and documentation. Collected concurrently with InspImgs, PathImgs contain an image sequence recorded continuously in time in order to capture the scene in front of the engineer as he/she walks through the building and thus document the path taken. Thus, PathImgs are not selectively captured by focusing on specific objects or damage, like how InspImgs are captured. PathImgs and InspImgs are synchronized in time, used for later localization. DrawImgs are part of the metadata captured using images [19]. DrawImgs contain a portion of a physical drawing while preserving its details. In the proposed technique, in addition to InspImgs and DrawImgs, which are normally captured in a typical reconnaissance mission, engineers simply collect PathImgs by mounting an extra camera on a hard hat or body.

After these images are gathered, in Step 2, the path of the engineer is reconstructed with the set of PathImgs using the DSO algorithm, which is one of the most popular VO algorithms (B). The 3D point cloud of the scenes included in PathImgs is also reconstructed. This process is to estimate relative locations between the PathImgs so that they are overlaid to the drawing, followed by localizing InspImgs. In this step, the structural drawing is also reconstructed from DrawImgs using the structure-from-motion algorithm (A). We previously developed the drawing reconstruction technique in reference [19]. 

Then, in Step 3, the path and 3D point cloud are overlaid onto the reconstructed drawing using a coordinate transformation (C). The transformation matrix is computed using an interactive tool by manually but rapidly finding the correspondence between a few images (around 10 images) in PathImgs (localized in the 3D point cloud) and their approximate capture locations on the drawing. Here, the purpose of mapping the path to the drawing is to localize InspImgs. Since the InspImgs are captured while PathImgs are continuously collected, approximate locations of InspImgs are easily identified from PathImgs having the closest timestamp (D). Additionally, PathImgs and InspImgs are used for reconstructing the local 3D textured model using the structure-from-motion algorithm (A). In the end, the user selects any InspImg and the proposed technique automatically informs its position on the reconstructed drawing and if needed, the scene on the selected InspImgs can be reconstructed in 3D. It is worth mentioning that the only manual process required in this technique is to manually match a select group of PathImgs with their corresponding locations on the drawing using the interactive tool for obtaining the transformation matrix.

### 3.1. Reconnaissance Image Collection

#### 3.1.1. Collecting InspImgs and DrawImgs

The procedure for acquiring InspImgs is governed primarily by typical protocols for obtaining reconnaissance images that are useful for documenting the perishable visual evidence of the hazard event. To gather high-quality images, InspImgs should be captured without blur. As the engineer approaches a building scene of interest, he/she aims to capture a few InspImgs of that specific scene from various perspectives and distances.

DrawImgs are independent of PathImgs and InspImgs and are meant to produce a high-resolution image of the structural drawing when that is not available in a digital or carriable form. Certain guidelines do need to be followed for taking DrawImgs, as explained in detail in [19]. In short, the physical drawing is placed in a flat and non-obstructed position; each DrawImg should have a large shared region (overlap), with adjacent DrawImgs; all contents of the drawing should be included in the complete set of DrawImgs; and, each DrawImg should be taken from a position in which the camera is pointing to the drawing while maintaining a similar distance from the drawing. 

#### 3.1.2. Collecting PathImgs

PathImgs are a sequence of images that are taken automatically by a compact and mountable camera that can be carried during the building walk-through. They record a stream of scenes that the field engineer observes along this path, and eventually, they are used for reconstructing the entire of the path using DSO, that the engineer walks through. Engineers do not need to take extra efforts to collect PathImgs if the camera is mounted on the hard hat or chest. Since PathImgs are collected mainly for this purpose, there is no need for the engineer to modify his or her motions or directions. Additionally, PathImgs are not the images that engineers will sift through for inspection or condition assessment. However, there are some considerations on the selection of the camera and its calibration before collecting images.

There are three factors to consider when the camera is selected. First, a global shutter camera is the best option for collecting PathImgs, while a rolling shutter camera would not be a suitable choice. This choice allows for avoiding the jello effect in PathImgs [39]. Second, motion blur in the PathImgs must be avoided. The selected camera should support a faster shutter speed with high ISO without dropping image quality. Third, the camera should support a high frame-per-second (fps) video or continuous shots. If the absolute motion between two consecutive PathImgs is too large, the DSO algorithm will produce large modeling errors and they are accumulated in the course of path reconstruction. These three considerations will guarantee the capture of valid PathImgs that can be used for accurate path reconstruction.

To use DSO, initial camera calibration is necessary. The intrinsic parameters of the compact camera must be determined accurately through the camera calibration process when they are not provided by the manufacturer. There are many ways to calibrate a digital camera, but the chessboard calibration method is widely used to find all these parameters [40,41]. In this method, a chessboard pattern having clean borders between black and white cells is placed on a flat table or attached to a wall, and the camera is used to take images from various angles with the full chessboard in view. Normally 10 to 30 calibration images are sufficient to perform geometric camera calibration [40,41]. This camera calibration is independent of the PathImg collection and should be done before the actual data collection.

### 3.2. Path Reconstruction

We adopted DSO to generate the path associated with data collection. The path was generated based on the stream of PathImgs gathered during the mission. Based on the performance evaluation described in the original DSO-based work and our preliminary tests, DSO has shown superior performance in terms of accuracy and speed among several monocular VOs. Additionally, DSO offers an easy-to-implement strategy and does not require special programming libraries and hardware. 

**Figure 2:**
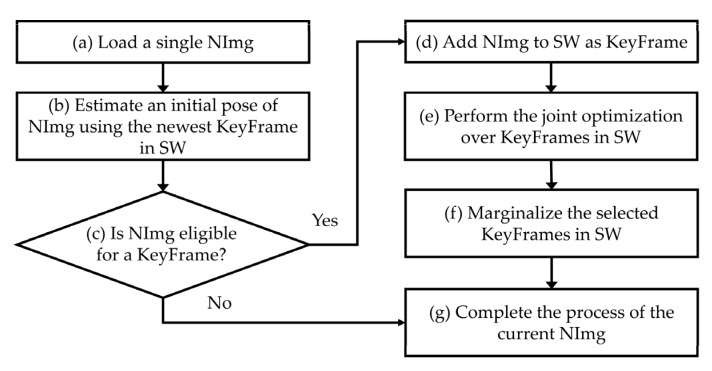
Flow chart of the direct sparse odometry (DSO) algorithm.

To better understand the working principle of DSO, the workflow of the algorithm is summarized in Figure 2 when a new image is inputted (hereafter, NImg) [38]. Steps (a)–(h) in the procedure were repeated for each subsequent NImg until all images (PathImgs) were scanned (inputted). Basically, the point cloud and pose estimation were performed on a subset of images, called the sliding window (hereafter, SW) and the images in SW are called key frames (hereafter, KeyFrames). The process in Figure 2 is to determine whether the NImg is eligible for KeyFrame and to perform the joint optimization over the KeyFrames to update the point cloud and the pose of the KeyFrames once the NImg is added to the SW. 

In Step (a), a single NImg is fed into the algorithm. Then, an initial pose of that NImg is roughly estimated by matching the points on the newest existing KeyFrame in SW. With the outcomes from Step (b), a series of strategies are applied to decide whether or not NImg can serve as a new KeyFrame in SW. This decision is based on, for example, i) whether or not the field of view has changed since the most recent KeyFrame, which is measured the mean square optical flow from the newest KeyFrame to NImg, ii) a camera translation has caused an occlusion or disocclusion, which is measured by the mean flow without rotation, or iii) the camera exposure time has changed significantly, which is measured by the relative brightness factor between the newest KeyFrame and NImg [38]. A condition for candidacy as a new KeyFrame is the image having a large relative movement from previous KeyFrames. In Step (c), if the NImg is not eligible for a new KeyFrame, the NImg only contributes to update the depth values of inactive points in SW, which are the points used for the future joint optimization. The NImg assigned as non-KeyFrame is not involved in the optimization process for point cloud and pose update. If NImg is qualified as the new KeyFrame, the NImg will be added into SW in Step (d). Subsequently, a joint optimization is performed, which is the core part of DSO in Step (e). The technical details of this process are delineated in the following paragraph. In short, the intensity difference between the points on the newly added KeyFrame and the existing KeyFrames in SW is minimized using the Gauss-Newton method for parameter optimization. Once the new KeyFrame is added in SW and successfully registered in the existing model, in Step (f), we deactivated some KeyFrames in SW, which will not contribute to the upcoming match with NImg. This process is called marginalization and helps to maintain a consistent number of KeyFrames in the SW, thereby improving the efficiency of the optimization in DSO. Finally, in Step (g) the operations related to the current NImg ends, and the same process is then repeated for the next NImg. 

In the optimization in Step (e), a cost function is designed to minimize the difference of intensity values between points in each KeyFrames with the projected points from all the other KeyFrames in SW. The formulation of the cost function starts with the difference between one point in the host KeyFrame and the projected point from a reference KeyFrame in SW, denoted $E_{pij}$ as

(1) $$E_{pij}:=\|I_{j}(p^{\prime })-I_{i}{\left(p)\right\|}_{2}$$

where the points from the host (i) and reference (j) KeyFrame are denoted as p and $p^{\prime }$, respectively. I stands for the pixel intensity and I(p) is the intensity value at p. In addition, $\|\cdot {\|}_{2}$ is the $l^{2}$-norm. Equation (1) computes the difference of intensity values between the point p and the projected point $p^{\prime }$. The process of projection described in [38] is based on a pinhole camera geometry as

(2) $$p^{\prime }=\Pi_{c}\left(R\cdot \Pi_{c}^{-1}\left(p,d_{p}\right)+t\right)$$

where the movement from the position where the camera takes KeyFrame i to KeyFrame j is modeled as a rotation and translation. R is a rotation matrix, and t is the translation vector. $d_{p}$ is depth value of point *p,* which is the perpendicular distance from the principal plane of the camera to the world point, which p represents in KeyFrame i. $\Pi_{c}()$ stands for the projection process of the camera from the corresponding world point to the image point p, and $\Pi_{c}^{-1}()$ is the inverse projection process.

The DSO algorithm considers an intensity calibration factor in Equation (1), expanding it to

(3) $$E_{pij}:=\underset{p\in N_{p}}{\sum }w_{p}\|I_{raw}\left(I_{j}\left(p^{\prime }\right)\right)-I_{raw}{\left(I_{i}\left(p\right)\right)\|}_{r}=\underset{p\in N_{p}}{\sum }w_{p}\|\left(I_{j}(p^{\prime }\right)-b_{j})-\frac{t_{j}e^{a_{j}}}{t_{i}e^{a_{i}}}{\left(I_{i}\left(p\right)-b_{i})\right\|}_{r}$$

To compensate for unknown intensity calibration factors involved in the imaging process of a camera, a function is defined to reflect such a process as

(4) $$I_{raw}\left(I\left(p\right)\right)=\frac{I\left(p\right)-b}{te^{a}}$$

This equation converts the intensity value of point p in one image to the raw intensity value that the camera should capture. Here, t is the exposure time of the image, a and b are constants regulating this converting process. These two parameters are taken as unknown values to be calibrated in the optimization. Note that if a camera does not record an accurate exposure time, the exposure time t is simply set to 1. Here, $l^{2}$-norm is replaced by the Huber norm $\|\cdot {\|}_{r}$ [42] to increase resistance to outliers. In addition, the weight term ($w_{p}$) in Equation (3) is used to accommodate points with different gradients. The weight term is defined in [38] as

(5) $$w_{p}=\frac{c^{2}}{c^{2}+{\left|\left|\nabla I_{i}\left(p\right)\right|\right|}_{2}^{2}}$$

where $\nabla I_{i}\left(p\right)$ is the gradient vector at point p in KeyFrame i, and ${\left|\left|\nabla I_{i}\left(p\right)\right|\right|}_{2}^{2}$ is the square of its $l^{2}$-norm. A factor c is a constant regulator and is set to 0.75 in our work. To improve the robustness of the cost function, DSO utilizes eight neighborhood points to compute the intensity difference for point p including its nearby region. This set of points is denoted as $N_{p}$.

$E_{pij}$ is summed up over the points in all host-reference KeyFrame combinations. $E_{pij}$ is established in terms of point p in KeyFrame i, which is observed in KeyFrame j. $\underset{j\in obs\left(p\right)}{\sum }$ is the summation where j over all the KeyFrames in SW in which p is visible. $\underset{p\in P_{i}}{\sum }$ is where point p over all the points $P_{i}$ in KeyFrame i. $\underset{i\in F}{\sum }$ indicates that i becomes all the KeyFrames in SW. Thus, the final cost function is defined as

(6) $$E=\underset{i\in F}{\sum }\underset{p\in P_{i}}{\sum }\underset{j\in obs\left(p\right)}{\sum }E_{pij}$$

The Gauss-Newton method is used to find the global minimum of this cost function. The unknown parameters to be computed include the rotation matrix R, the translation vector t, the depth value $d_{p}$ of point p, parameters of the imaging process a and b, and the camera intrinsic parameters [38]. The camera intrinsic parameters are treated as variables in the optimization for fine-tuning from the initial estimates found in the camera calibration. Among these parameters, the camera position, R and t, are the desired output in our study. 

### 3.3. Drawing Reconstruction

Images of structural drawings are often collected as a part of a building reconnaissance dataset to document the details of the structural system and design. When the digitized version of the drawings is not available, for example with older buildings, field engineers must take multiple photographs of the hard copy of the structural drawings to capture this information in a legible and complete form. To accomplish this task, they capture DrawImgs, because it is often difficult to include the entire view in one single photograph. A method is available to automatically organize these DrawImgs and restore a complete high-resolution drawing in a digital form from these DrawImgs by the coauthors. DrawImgs are first automatically filtered out from the entire building image collection using a convolutional neural network classifier. Then, the DrawImgs are grouped according to the original drawing that they belong to. After that, a full reconstruction of each page of the drawings is obtained. More details are provided in [19]. 

### 3.4. Overlaying the Path with the Drawing

The 3D point cloud is projected to a 2D plane in the gravity (height) direction. Thus, we could use two independent sets of 2D points to represent the path defined by the 3D point cloud and the reconstructed drawing. Then the path can be overlaid onto the drawing by finding the transformation matrix between these two sets of data. Based on the correspondence between the locations of some PathImgs and their locations on the drawing, the transformation matrix for projecting 3D points cloud and path onto the drawing can be computed. We used the absolute orientation method to find the optimal transformation matrix between these correspondences [43].

**Figure 3:**
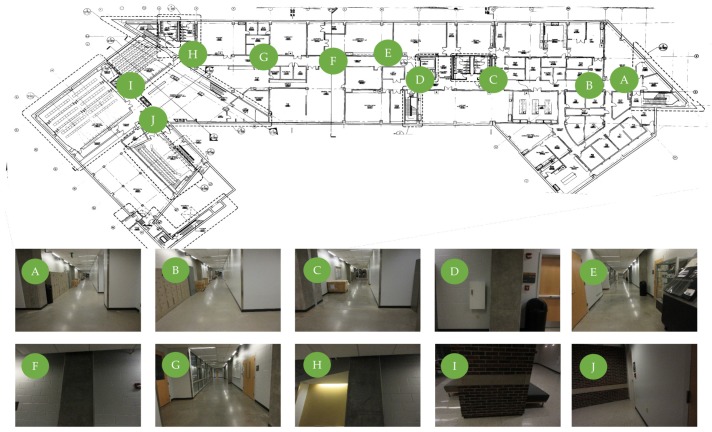
Path transformation: 10 images are matched with the corresponding locations on the reconstructed drawing.

An interactive tool was developed to assist with this task. The objective of this tool is to rapidly match PathImgs with their locations on the reconstructed drawing. The tool shows a group of PathImgs to the engineers so that they can select and match PathImgs to the corresponding locations on the reconstructed drawing. If there is no suitable PathImg in the group, or if the location is hard to recognize, the engineers can select PathImgs from another group. The tool supports the function of enlarging the drawing to improve selecting the location. We repeated this process until engineers have selected a sufficient number of image-location pairs. Around 10 pairings across the drawing are sufficient for obtaining the transformation matrix. The more pairings are given, the more accurate the projection result will be. Note that the selected points should be equally distributed over the entire path rather than gathered in a specific region of the drawing. Once the selection process is completed, the tool automatically computes the transformation matrix and conducts the coordinate transformation to overlay the path and 3D point cloud onto the drawing as the outcome. Figure 3 shows 10 PathImg-locations pairs used for experimental demonstration in Section 4. The images on the right are PathImgs selected and their corresponding locations are marked on the reconstructed drawing.

## 4. Experimental Verification

### 4.1. Description of the Test Site

**Figure 4:**
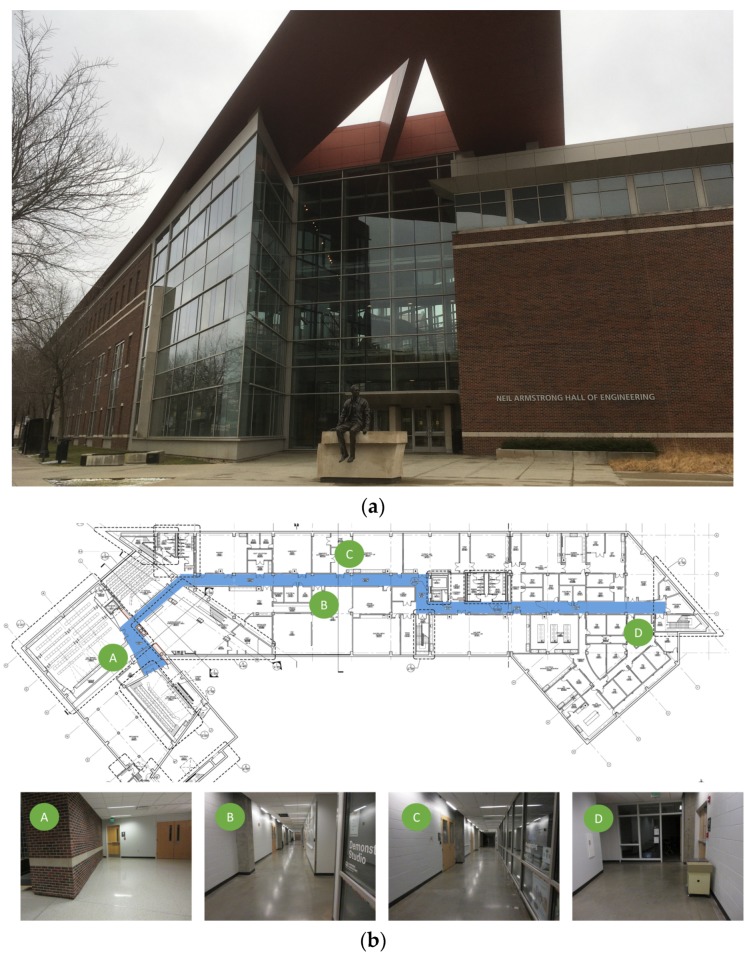
Experimental test site (Armstrong Hall at Purdue University, United States): (**a**) building overview and (**b**) basement floor plan: InspImgs and PathImgs are collected along the corridor highlighted as solid blue. Sample images corresponding to key spots in the corridor are provided.

Experimental verification was performed on an actual building. We chose the basement floor of Armstrong Hall on the Purdue University campus as a test site, shown in Figure 4a. The area of the basement floor was about 175 m × 60 m and its digital drawing including sample images of key places is presented in Figure 4b. A long corridor of 175 m (long) by 3 m (wide) was located along the centerline of the floor, marked as solid blue in Figure 4b. Since the corridor was sufficiently long and had several turns to walk through, and the structural columns were exposed in the corridor (see Figure 4b), the scenes in the test site do represent the actual building environment that engineers would visit after earthquake events. We collected necessary images including InspImgs and PathImgs by emulating inspection and data collection steps taken in a typical post-earthquake field reconnaissance mission. 

### 4.2. Collection of the Image Data

**Figure 5:**
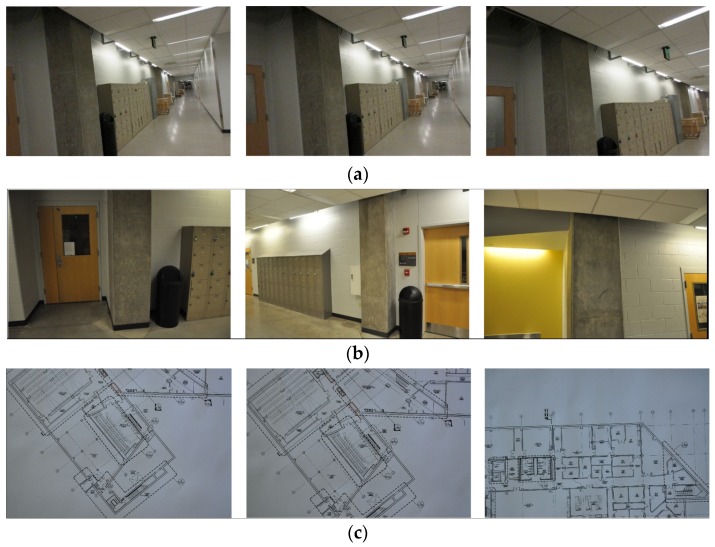
Sample images collected during the test: (**a**) PathImgs, (**b**) InspImgs, and (**c**) DrawImgs

We manually collected PathImgs using a compact camera (Canon 350HS), InspImgs and DrawImgs using a DSLR camera (Nikon D90). The size of the Canon 350HS was 3.92 inches × 0.9 inches × 2.28 inches, and the weight was 5.19 ounces. Overall, 3687 PathImgs, 232 InspImgs, and 44 DrawImgs were collected, and their resolutions were 2595 pixels × 1944 pixels, 4288 pixels × 2848 pixels, and 4288 pixels × 2848 pixels, respectively. Their sample images are shown in Figure 5. The compact camera was set to burst mode, which continuously took PathImgs at 7.8 fps. The focal length was fixed throughout the entire experiment because the initial calibration parameters remained unchanged. We avoided the jelly effect in this experiment by simply walking slowly, at about 1/3 of the normal walking speed of a human. By doing this, we did not notice an obvious jelly effect, and if there were, the errors did not influence the quality of the result. Additionally, the Canon 350HS is just one sample of a suitable camera to collect PathImgs and a baseline for choosing the camera device. By using cameras with faster shutter speeds with high ISO, one can avoid the need to walk slower. In this experiment, two people collect InspImgs and PathImgs at the same time. However, the actual collection of PathImgs is devised to be automated with a mountable camera (e.g., action camera) by a single engineer. The DSLR camera was used to collect InspImgs and DrawImgs. A major distinction between InspImgs and PathImgs is that PathImgs represent a stream of image sequence without gazing at any specific objects or regions that the engineers would be interested in, while InspImgs are non-periodic image shots targeting objects-of-interest at various viewpoints and distances. For instance, assume that the engineer gazes at interesting objects (e.g., columns and walls) or certain evidence of damage (e.g., crack and spalling), PathImgs and InspImgs capture different information: PathImgs, as in Figure 5a, captures the views in front of the engineer, regardless of whether the compact camera is directly facing any particular building components of interest. The scenes in these images will turn upwards or downwards following the gaze of the engineer. On the other hand, InspImgs, as in Figure 5b, were aimed at the objects and regions that the engineer found interesting and chose to document. 

The image data collected was intended to cover the entire corridor area highlighted in Figure 4b. We followed the image collection guideline introduced in Section 3.1. The compact and DSLR cameras were set to have the same timestamp before conducting the experiment. We spent 11 min and 45 s to collect both InspImgs and PathImgs by walking through the entire corridor and performing inspection actions, such as observing structural conditions and taking more photos of structural elements, which is to emulate an actual post-earthquake reconnaissance mission. 

Regarding DrawImgs, we assumed the situation where only a paper copy of the drawing was available to the engineers (although in this case we did have a digital drawing shown in Figure 4b). The digital drawing was printed on a large engineering paper (A1) and DrawImgs captured the drawing, following the image collection guideline provided in the coauthor’s paper [19]. The paper copy of the drawing was placed on a large flat table and images were taken at a suitable distance from the drawing in such a way that details of the drawing (e.g., line and number) were visible. The total number of images depends on the size and details of the drawings. In this work, we collected 44 DrawImgs from a single drawing of the basement to capture all the details and some sample images are in Figure 5c. Note that the original technique proposed by the coauthor also performs image classification and drawing matching techniques so that DrawImgs are automatically extracted from a set of images collected and individual drawing images are created from DrawImgs captured from multiple drawings. However, in this work, we only implemented the drawing image generation (stitching) technique using a set of DrawImgs collected from a single drawing.

We used a workstation with an Intel i9-7920x CPU, 32 Gb memory, and a NVIDIA GeForce RTX 2080Ti video card. The path reconstruction with 3687 PathImgs and drawing reconstruction with 44 DrawImgs took less than 20 min. Generating the local 3D surface model for one scene took about 2.5 h using 402 images, although the actual time would vary for each case depending on the number of images. All these processes were fully automated. The only manual task was to match PathImgs to the corresponding locations in the reconstructed drawing in order to compute the transformation matrix between the 3D point cloud and the reconstructed drawing image. However, this task took less than five minutes to match 10 PathImgs, shown in Figure 3. 

### 4.3. Results

#### 4.3.1. Path Reconstruction

The path was reconstructed using a stream of PathImgs. Video footage was also applicable after transferring video footage to images. We used the compliable source code [44], published by the DSO creators, to generate the DSO software. It is written in C++, run in Linux 14.04, and operated with Linux bash command lines. When DSO was applied to PathImgs, KeyFrames were automatically extracted and used to estimate the positional information. Here, 1428 images were identified as KeyFrames and their relative positions in the reference coordinate were estimated. We linearly interpolated between every two consecutive KeyFrames, each PathImg was assigned with a relative position for both KeyFrames and non-KeyFrames, excluding PathImgs that were dropped in the initialization process. The reconstructed path consisted of a set of 3607 discrete points associated with PathImgs, and each discrete point had a 7-dimensional positional vector including a three dimensional translation and a four dimensional quaternion to represent a 3D rotation with respect to the reference coordinate system (as mentioned in Section 3.2). Moreover, the 3D point cloud was also generated from the scenes including wall, doors, columns, of which scenes were contained in KeyFrames. 

**Figure 6:**
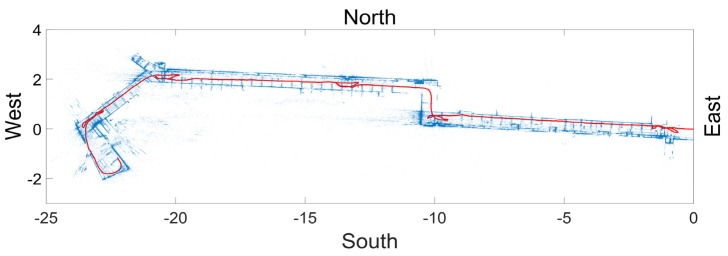
Reconstructed image collection path and a point cloud of the scenes on PathImgs: The points in blue represent the reconstructed point cloud and form a layout of the walls in the corridor. A set of red points passing through the corridor is the locations of PathImgs. The values are represented using a hypothetical unit in the initial reference coordinate system and have no physical scale information.

Figure 6 shows the reconstructed path and 3D point cloud, which were viewed (or projected) in the gravity (height) direction. Most PathImgs were normally captured while the gravity direction was aligned with the image height. Thus, we could easily compute the gravity direction of the reconstructed model for projection. In Figure 6, each red point indicates each PathImg location (although they look as if they were connected) and blue points were the point cloud (for a black and white figure, the line passing through the middle of the encompassed region was the set of red points.) A majority of points (blue points) were likely generated from the perpendicular features adjacent to the corridors like walls or doors. Thus, the blue points formed a layout of the walls along the corridor. 

As mentioned in Section 2 and Section 3.2, DSO was based on image collection using a monocular camera, which could not determine a real-world scale. Thus, the points in Figure 6 are represented in hypothetical units, which have no physical scale information. However, they are proportional to real-world units, facilitating mapping the path to the drawing image. Note that we manually rotated the reconstructed model in Figure 6 to be horizontal for better visualization.

#### 4.3.2. Drawing Reconstruction

**Figure 7:**
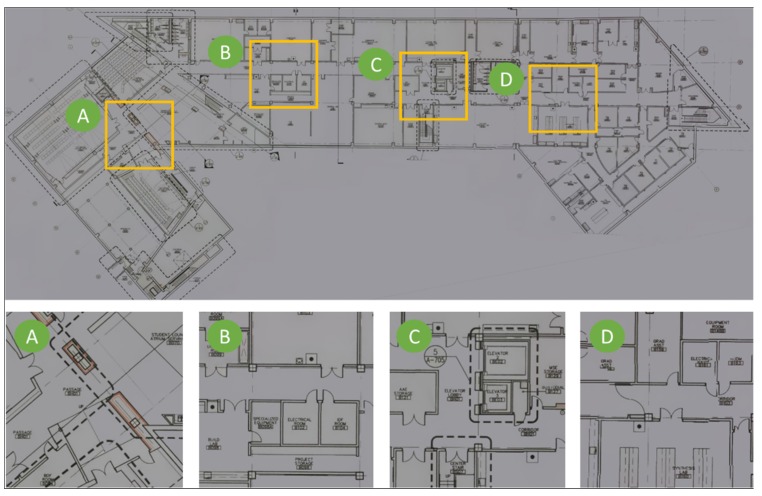
Drawing image reconstructed using DrawImgs: The four images on the bottom are magnified areas corresponding to the boxes on the drawing on the left.

The drawing reconstruction method is implemented to the collected DrawImgs and the resulting reconstructed image of the structural drawing is present in Figure 7. The overall quality of the drawing was quite satisfactory, and as was clear from the enlarged areas next to the full drawing. All detailed were preserved, even small texts and thin lines. The color and orientation of the reconstructed drawing in Figure 7 were manually tuned for better visualization. It should be emphasized that this method possessed the ability to automatically restore multiple drawings from a mixed set of DrawImgs that included images of more than one drawing. However, in this experiment, we only reconstructed a drawing for a single basement floor using the corresponding DrawImgs. Additionally, if the digital drawing is available, such a drawing reconstruction step can be skipped. This digital drawing was used directly for the overlay step as in the next section, replacing the reconstructed drawing image.

#### 4.3.3. Path Overlay

**Figure 8:**
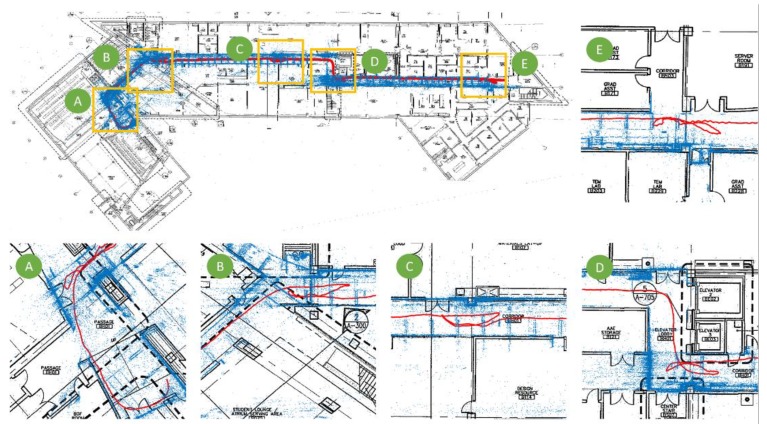
The reconstructed drawing in Figure 7 overlaid with the 3D point cloud and PathImgs locations in Figure 6.

Following the steps explained in Section 3.4, we selected 10 PathImgs and their corresponding positions in the reconstructed drawing for computing the transformation matrix. The transformation matrix was used to map the point cloud and PathImg locations (in Figure 6) to the reconstructed drawing (in Figure 7). The reconstructed drawing overlaid with the point cloud is shown in Figure 8. The overall result was quite accurate. A majority of blue points were well aligned with the corridor wall boundary on the drawing. Note that we did not conduct a quantitative evaluation of the mapping result because it was sufficient to identify approximate locations of PathImgs for the purpose of documenting the path of the engineer and associating specific images with that reconstructed path and the structural drawing. Here, the reconstructed drawing image in Figure 8 was the binary image converted from the drawing image in Figure 7 so that the line and text in black were clearly legible. 

In Figure 8, five regions on the drawing were enlarged. The walking (inspection) path (a set of red points) was not straight because we mimicked the actual inspection procedure, such as wandering around to see components-of-interest for close-up inspection. This type of walking is likely application-specific and certainly challenges our technique. Additionally, more blue points were generated near the scenes-of-interest because the inspector spent more time near those regions, collecting more PathImgs. 

### 4.4. Image Localization and Local 3D Textured Model Reconstruction

**Figure 9:**
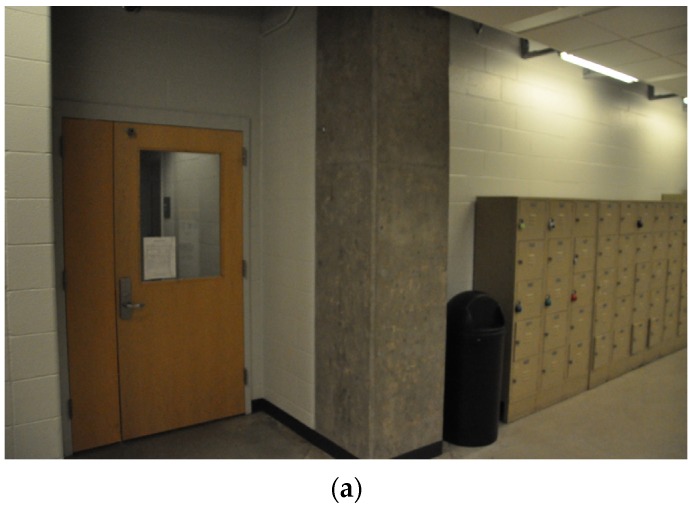

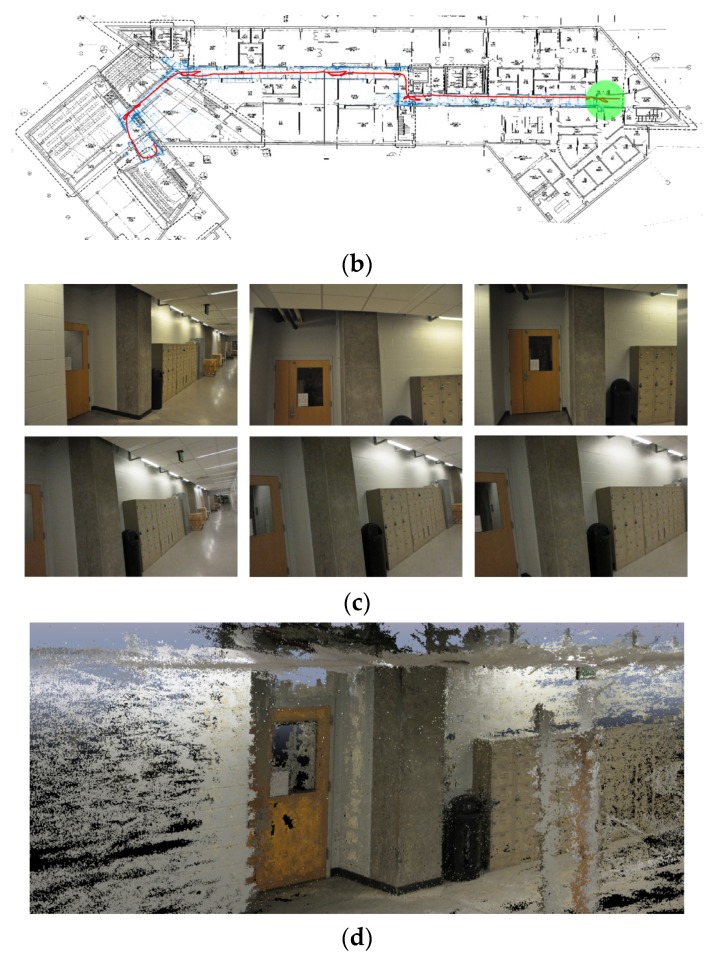
Image localization and local 3D textured model generation: (**a**) selected InspImg, (**b**) its location on the reconstructed drawing, (**c**) InspImgs (first row) and PathImgs (second row) collected at a similar time when the selected InspImg is taken, and (**d**) reconstructed local 3D textured model.

We made an in-house tool for reviewing InspImgs and their localization results using MATLAB. When the user selects a particular InspImg, the tool searches for the PathImg that is captured around the same time when the selected InspImg was taken. Then, the location of the selected InspImg is automatically marked on the drawing. For example, Figure 9a shows a selected InspImg, which contains a reinforced concrete structural column. Figure 9b is the location of the corresponding image automatically marked as a circle on the reconstructed drawing. 

In the method, we did not combine the tool with an SfM-based 3D texture modeler. Herein, we simply show the capability for generating a 3D textured model using SfM software. As mentioned in Section 4.2, the clocks of both the compact and DSLR cameras were synchronized. Thus, the selected InspImg could be readily paired with the PathImgs and InspImgs that have a nearby timestamp. We roughly set 30 s for PathImgs and 5 s for InspImgs, both before and after, to extract views containing the same scene on the selected InspImg. This set of extracted images became an input for the SfM software. Here, for this task only, we used the commercial software, Pix4D mapper 4.4.4. In Figure 9c, samples of identified PathImgs and InspImgs are shown. These samples were captured during a time range around the selected image in Figure 9a. The local 3D textured model generated using the images in Figure 9c is shown in Figure 9d. Once a user selects any InspImg, the full process, including its localization and local 3D textured model construction, is automated. Then, engineers can review the scenes on the selected InspImg with sufficient spatial context. 

## 5. Conclusions

In post-event building reconnaissance, the collection of hundreds of images from a single building to preserve valuable evidence and document its condition is becoming a common process. However, when such images are taken in an indoor environment, it is difficult to recall and document where each image was taken. Without spatial information, engineers are not able to conduct in-depth studies needed to understand the consequences of natural hazard events on our buildings and improve our building codes. To address this issue, we developed a technique to automatically localize inspection images on a drawing image and generate a local 3D textured model of a scene-of-interest. The technique does not require extra effort as compared to the existing data collection procedures. Data collection does not require the use of expensive cameras or special 3D sensors. An engineer can simply carry an extra compact camera, potentially mounted on one’s hard hat or body, to gather the additional images needed for localizing the inspection image. If using a camera with low fps (~7.8 fps in this work), the engineer needs to reduce the walking speed to about 1/3 of the normal speed. Additionally, if the camera possesses a faster fps with high ISO without dropping the image quality, this limitation can be lifted. Direct sparse odometry is used to reconstruct the path of data collection and the 3D point cloud of the scenes. The reconstructed path and point cloud are then projected onto the structural drawing images so that inspection images can be localized. Once these images are collected, the process is fully automated except for manually matching a few images to compute the transformation matrix between the reconstructed path and the drawing image. Moreover, using the images collected for path reconstruction and inspection, a local 3D textured model of the scene-of-interest can be reconstructed using the structure-from-motion algorithm. The capability of the technique was fully demonstrated using the experimental study conducted on the actual building. This technique provides comprehensive and detailed spatial information of the images collected in the field, thus facilitating a much broader range of studies using image data.
